# Pregnancy in Takayasu Arteritis: A Case Report of Successful Pregnancy in a Large Aortic Aneurysm

**DOI:** 10.1002/ccr3.70974

**Published:** 2025-10-01

**Authors:** Mona Yadollahi, Simin Almasi, Mehrdad Salehi, Farnoosh Larti, Mahdi Daliri, Akram Sardari

**Affiliations:** ^1^ Rajaie Cardiovascular Medical and Research Center Iran University of Medical Sciences Tehran Iran; ^2^ Department of Rheumatology, Firoozgar Hospital Iran University of Medical Sciences Tehran Iran; ^3^ Cardiothoracic Surgery Department, Imam Khomeini Hospital Complex, School of Medicine Tehran University of Medical Sciences Tehran Iran; ^4^ Cardiology Department, Imam Khomeini Hospital Complex, School of Medicine Tehran University of Medical Sciences Tehran Iran; ^5^ Heart Valve Disease Research Center Rajaie Cardiovascular Medical and Research Center Tehran Iran

**Keywords:** ascending aorta aneurysm, bentall operation, pregnancy, takayasu arteritis

## Abstract

A 29‐year‐old primigravida woman with a gestational age of 19 weeks visited in the cardio‐obstetrics clinic for hypertension. Physical examination revealed auscultated systolic and diastolic murmurs in the second right intercostal space. Transthoracic echocardiography (TTE) showed severe left ventricle (LV) enlargement with mild systolic dysfunction. Severe aortic regurgitation (AR) and aneurysmal dilation of the proximal part of the ascending aorta were noted. Magnetic resonance imaging (MRI) without contrast showed a dilated aortic root and ascending aorta (64.4 mm at the right pulmonary artery level). Imaging and serologic findings were compatible with acute Takayasu arteritis. Despite the detailed discussion, the patient refused surgery. During the hospital admission, the patient remained stable with no change in the size of the ascending aorta. Cesarean section was done at 32 weeks of gestation. A boy with a body weight of 1360 g was born. 10 days after delivery, she underwent a bio‐Bentall procedure. Follow‐ups of the patient and her child were uneventful 12 months after delivery.

**Summary**

Takayasu arteritis (TA) in pregnant women with hypertension and evidence of inflammation or evidence of pulse deficit should be considered. If the main manifestation of the disease is the dilatation of the aorta, the effect of pregnancy on its course cannot be predicted. In case of the active TA and severe dilation of aorta, individual decision making with termination of pregnancy and aorta surgery should be considered.

## 1 Introduction

Takayasu arteritis (TA) is a nonspecific large‐vessel inflammation that primarily affects the aorta and its branches. The impact of pregnancy on the TA course has not been proven. Data on the best management for aortic aneurysms in pregnant patients with TA are limited. There is a case report of a stable dilated ascending aortic aneurysm during pregnancy.

## 2 Case History

A 29‐year‐old primigravida woman with a gestational age of 19 weeks was visited for hypertension in the cardio‐obstetrics clinic. She had no complaint of dyspnea, fatigue, palpitation, or chest pain. Physical examination revealed a wide pulse pressure, with blood pressure readings of 148/52 mmHg in the right arm and 152/50 mmHg in the left arm. Systolic and diastolic murmurs in the second right intercostal space were auscultated.

## 3 Investigations and Treatment

**FIGURE 1:**
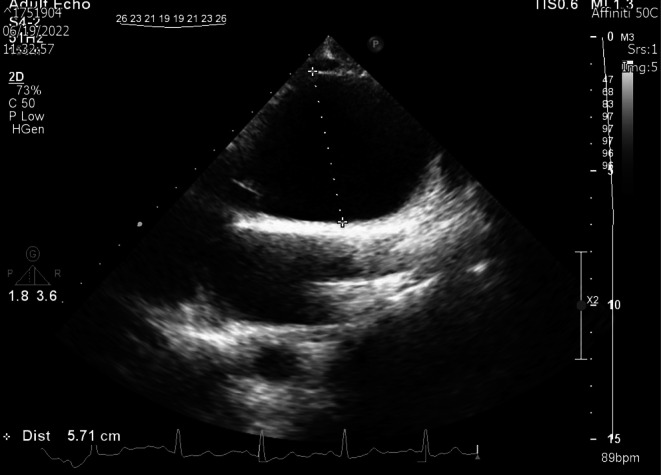
Para‐sternal long‐axis view of transthoracic echocardiography showing the size of the ascending aorta during pregnancy.

Transthoracic echocardiography (TTE) showed severe left ventricular (LV) enlargement with mild systolic dysfunction (LV ejection fraction about 52% by biplane Simpson's method). Severe aortic regurgitation (AR) and aneurysmal dilation of the proximal part of the ascending aorta (57 mm) were noted (Figure 1). In TTE, the ascending aorta had a relatively thickened wall, and narrowing of the abdominal aorta was detected.

The results of the laboratory tests were as follows: erythrocyte sedimentation rate (ESR) 90 mm/h, C‐reactive protein (CRP) 30 mg/L, and white blood cell (WBC) count 11,100/mm^3^ with 70% neutrophils. Evidence of normochromic normocytic anemia with a hemoglobin (Hgb) level of 10.7 g/dL and a platelet count of 303,000/mm^3^ was also found. Blood culture, Coombs Wright, 2ME, anti‐cardiolipin Ab, ANCA tests, and complement analysis were negative.

**FIGURE 2:**
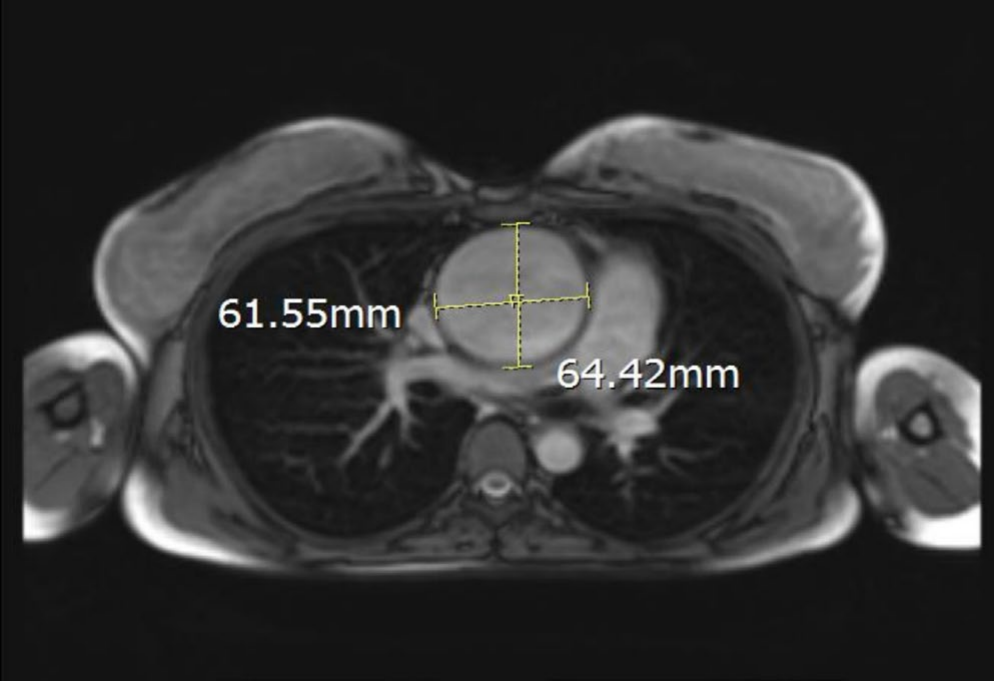
Axial view of thoracic magnetic resonance image without contrast during pregnancy.

Color and pulse duplex study of the abdominal aorta showed mild narrowing in the infra‐renal portion of the aorta with mildly increased velocity. Magnetic resonance imaging (MRI) without contrast was performed as another modality for further evaluation of the aorta. Dilated aortic root and ascending aorta (64.4 mm at the right pulmonary artery level) with thickening of the ascending aorta wall (about 4 mm), inflammation, and edema in the descending and abdominal aorta, with significant stenosis of the infra‐renal region were reported (Figure 2). Doppler study of umbilical vessels showed increased resistance.

Imaging and serologic findings were compatible with acute TA, and after consultation with the rheumatologist, medical treatment and surgery for the aortic aneurysm and termination of pregnancy were proposed. Despite the detailed discussion, the patient refused surgery for the fetus's safety and continued the pregnancy. After passing the official process and filling out a written consent, pregnancy was continued under close supervision with serial TTE to monitor the size of the ascending aorta.

She received a pulse of methylprednisolone and 3 mg/kg infliximab and was discharged with oral prednisolone. Until 29 weeks of pregnancy, the size of the aorta remained unchanged. At this time, the patient was referred to a tertiary cardio‐obstetrics center for vigilant monitoring of the aorta and proceeding to the termination of pregnancy and urgent cardiac surgery upon the patient's condition.

**FIGURE 3:**
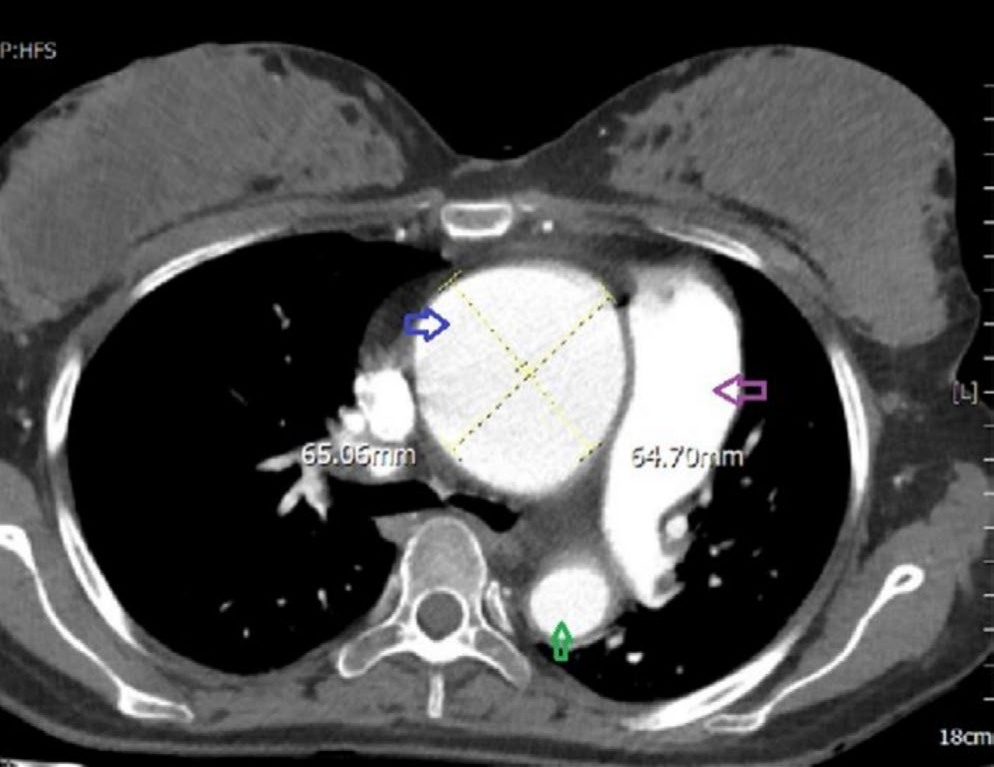
Axial view of thoracic aorta CT angiography after pregnancy. Blue arrow: aneurysm of ascending aorta; green arrow: descending thoracic aorta; purple arrow: main pulmonary artery trunk.

During the hospital admission, the patient remained stable with no change in the size of the ascending aorta. Cesarean section was done at 32 weeks of gestation. A boy weighing 1360 g was born. After delivery, CT angiography of the aorta was done. Aneurysmal ascending aorta dilation (65 mm) was seen (Figure 3). As laboratory data showed persistent inflammation, a second course of corticosteroids and anti‐TNF therapy was prescribed.

Regarding the size of the aneurysm and persistent evidence of inflammation, cardiac surgery was performed 10 days after delivery. During the surgery, the aortic valve leaflets were resected, and both the left main (LM) and right coronary artery (RCA) were released. The aortic valve, ascending aorta, and its root were replaced with a biological valve (Resilia, size 23) and a tubular graft (size 28). Then, LM and RCA were implanted on it, and a distal anastomosis of the tubular graft to the native aorta was performed end‐to‐end.

## 4 Conclusions

We reported the pregnancy course and management of a pregnant woman with TA, ascending aorta aneurysm, and severe AR. The pregnancy course was uneventful, and a cesarean section was done at 32 weeks of gestation. After delivery, the bio‐Bentall procedure was performed without any complications. Follow‐ups of the patient and her baby were uneventful 12 months after delivery.

## 5 Discussion

TA is a nonspecific large‐vessel inflammation that primarily affects the aorta and its branches. It is more common in women younger than 40 years of childbearing age [1].

TA commonly leads to stenosis and fibrosis in more than 90% of patients. During the severe and acute inflammation phase, aneurysm formation occurs in approximately 25% of patients [2].

There exists an established criterion for TA, including age and gender, elevated inflammatory markers, and new features of aortic involvement in the daily clinic [3].

The impact of the pregnancy on the TA course is not proven [4], but maternal and fetal complications are seen in a patient with TA during pregnancy [5]. The most frequent complication in pregnancy is maternal hypertension, with variable frequency and direct effect on fetal outcome [6].

New onset or worsening maternal hypertension, renal artery involvement, heart disease, and active inflammation are correlated with unfavorable pregnancy consequences and maternal complications [7].

Cardiovascular and cerebrovascular events are the major causes of maternal mortality in TA [7].

A recent systematic review and meta‐analysis of pregnant women with TA showed an increased risk of hypertension, miscarriage, and preeclampsia in these women during pregnancy. If conception is achieved despite low disease activity, the outcome is better. Advanced maternal age and prolonged disease duration did not increase maternal complications [8].

A dedicated examination of women with hypertension during pregnancy is mandatory. In our patient, hypertension was the presenting feature of TA discovered during pregnancy. Moreover, managing the active phase of the TA was complicated by the aneurysmal dilation of the ascending aorta. In TA, the ascending aorta is the most common site of aneurysmal dilation, with a frequency of about 33% [9]. Considering the fibrosis and scar formation of the intima and adventitia, dissection is a rare consequence of this arteritis [10].

Data on the best management for aortic aneurysms in pregnant patients with TA is limited. There is a case report of a stable abdominal aortic aneurysm with a size of 47 mm during pregnancy [11]. In another report, the surgery of a 58 mm ascending aorta aneurysm in a woman with TA was done during the 19th week of pregnancy [12]. The uncertainty in applying the best treatment options for aortic aneurysms due to TA in pregnancy necessitates multidisciplinary care. Concomitant vigorous treatment of hypertension is recommended [9]. Aneurysm management depends on the severity of the aneurysm, its location, and its size. Surgery during the active phase of the TA increases surgical mortality; therefore, interventions should be performed in the remission phase whenever possible [13].

Corticosteroid therapy is the most effective treatment for controlling inflammation in TA and reduces perioperative complications. In complex and refractory patients, anti‐TNF and other biologic agents are utilized. Although surgery remains the ultimate treatment for aortic aneurysms [14, 15].

## Author Contributions

**Mona Yadollahi:** conceptualization, data curation, writing – original draft, writing – review and editing. **Simin Almasi:** data curation. **Mehrdad Salehi:** data curation. **Farnoosh Larti:** writing – review and editing. **Mahdi Daliri:** writing – original draft. **Akram Sardari:** conceptualization, data curation, writing – original draft, writing – review and editing.

## Ethics Statement

Ethical approval was obtained from Rajaie Cardiovascular Medical and Research Center, Iran University of Medical Sciences, under the code 1403.061.

## Consent

Written informed consent was obtained from the patient to publish this report in accordance with the journal's patient consent policy.

## Conflicts of Interest

The authors declare no conflicts of interest.

## Data Availability

All data generated during this study are available from the corresponding author upon request.
